# Tactile Method in Confirming Proper Endotracheal Intubation in Emergency Setting; a Letter to Editor

**DOI:** 10.22037/aaem.v9i1.1148

**Published:** 2021-05-09

**Authors:** Behrang Rezvani Kakhki, Mohsen Miri, Morteza Talebi Doluee, Zeynab Sabeti Baygi, Zahra Abbasi Shaye, Elnaz Vafadar Moradi

**Affiliations:** 1 Department of Emergency Medicine, Faculty of Medicine, Mashhad University of Medical Science, Mashhad, Iran.; 2 Department of Obstetrics and Gynaecology, Faculty of Medicine, Mashhad university of Medical Science, Mashhad, Iran.; 3 Clinical Research Development Unit, Faculty of Medicine, Mashhad University of Medical Sciences, Mashhad, Iran.

**Dear Editor,**

Orotracheal intubation is one of the sure ways to manage airways in critical patients (1, 2). Failed intubation (Failure to properly place the endotracheal tube (ETT) in trachea) is a rather common event (3). There have been many techniques to confirm proper intubation, but none of them are applicable in all conditions. Methods such as capnography, tracheal sonography and chest-X-ray, were introduced for verification of proper tracheal intubation but they have their own limitations (4, 5). Given the significance of proper airway management, the authors focused on a secondary method of verifying proper intubation using tracheal tactile method and compared it to existing methods. 

This cross-sectional study was conducted on patients in need of intubation at Emergency Departments of Emam Reza, Ghaem, and Hasheminejad Hospitals of Mashhad, Iran. Patients with abnormal airway anatomies, cardiopulmonary arrest, severe cervical trauma, and tracheal or endobronchial traumas with increased risk of aspiration were excluded. Ethics Committee of Mashhad University of Medical Sciences approved the protocol of this study (Ethics code: IR.MUMS.fm.REC.1395.644). Orotracheal intubation was performed for all participants and at the same time a well-trained expert (resident) touched the trachea (thumb and index finger on both sides of the trachea under the Adam’s apple) without applying any pressure. When the tube was inserted, he/she would tell whether he/she thought the tube was properly placed. Other than tactile method, capnography, chest auscultation, and chest-X-ray (all 3 measures) were performed for confirmation of ETT placement (as gold standard) in all cases. Cases in which 2 out of 3 methods, one of which was always capnography, confirmed the proper location of ETT, were considered as proper intubation. 

Finally, 181 patients with mean age of 71.2 ± 16 years were studied (58.8% male). Based on the tactile method, in 161 (89%) cases, the tube had passed into the trachea. However, other methods confirmed proper intubation in 171 (94.5%) cases. Sensitivity, specificity, and positive and negative predictive values of tactile method in confirmation of proper placement of endotracheal tube were 93% (95% CI: 88%-95%), 80% (95% CI: 76%-83%)), 98.7% (95% CI: 96%-100%), and 40% (95% CI: 38%-44%), respectively. In addition, positive and negative likelihood ratios of tactile method in this regard were 4.65 and 0.087, respectively. With the tactile method, it was observed that there were 159 cases of true positive and 12 cases of false positive, which could be due to shortage of time and poor technical skills due to little experience in the tested method. It seems that, the method may perform much better with more time and practice.

Our results support the results of Gamble et al. performed on 50 children between 2 and 10 years old, who were divided into 3 groups based on tactile confirmation of ETT placement through their suprasternal notch (6). Eventually, they concluded that the tactile method provided better clinical results than Pediatric advanced life support (PALS) formula in guiding intubation. 

McKay et al. studied 77 people and observed that tactile ETT tube placement confirmation via the suprasternal notch was easy or relatively easy in 60 cases, and hard or impossible in 17. They concluded that higher age, diabetes and smoking were associated with rigidity in tracheal rings, which prevents a good sense of touch on the intubation site (7). Their results match those of the present study with one difference; the effects of underlying factors on verification of tracheal intubation were not evaluated in the present study, but will hopefully be the subject of future studies.

According to the authors’ findings, it could be concluded that the tactile technique is a quick, inexpensive, accurate and risk-free technique to verify proper ETT placement without any need for special equipment or skills. 
